# Clinical Differences and Non-Alcoholic Fatty Liver Disease-Related Factors of Lean and Non-Lean Patients with Metabolic Syndrome

**DOI:** 10.3390/jcm11092445

**Published:** 2022-04-26

**Authors:** Punyisa Boonchai, Chayanis Kositamongkol, Suchanart Jitrukthai, Sukumal Phothirat, Euarat Mepramoon, Pongpol Nimitpunya, Weerachai Srivanichakorn, Thanet Chaisathaphol, Chaiwat Washirasaksiri, Chonticha Auesomwang, Tullaya Sitasuwan, Rungsima Tinmanee, Naruemit Sayabovorn, Phunchai Charatcharoenwitthaya, Pochamana Phisalprapa

**Affiliations:** 1 Division of Ambulatory Medicine, Department of Medicine, Faculty of Medicine Siriraj Hospital, Mahidol University, Bangkok 10700, Thailand; punyisaamm1906@gmail.com (P.B.); chayanis.kos@mahidol.edu (C.K.); suchanart.jit@gmail.com (S.J.); sukumal.pho@student.mahidol.edu (S.P.); euarat.me@gmail.com (E.M.); pongpol.nim@gmail.com (P.N.); pop.weerachai@gmail.com (W.S.); thanet_bin@yahoo.co.th (T.C.); golf_si36@hotmail.com (C.W.); chonticha_nui@yahoo.com (C.A.); tullaya.sita@gmail.com (T.S.); annrungsima@gmail.com (R.T.); naruemit114@gmail.com (N.S.); 2 Division of Gastroenterology, Department of Medicine, Faculty of Medicine Siriraj Hospital, Mahidol University, Bangkok 10700, Thailand; phunchai@yahoo.com

**Keywords:** Asian, liver fibrosis, metabolic syndrome, non-alcoholic fatty liver disease, non-invasive scoring system, transient elastography, ultrasonography

## Abstract

This study investigated differences in the clinical data and prevalence of lean and non-lean patients with non-alcoholic fatty liver disease (NAFLD) and metabolic syndrome (MetS). Data on patients with MetS who had results of ultrasonography or transient elastography were collected from a Thai university hospital database. Patients with exclusion criteria for NAFLD diagnosis were excluded. Patients’ clinical characteristic and the performances of three non-invasive scoring systems (fatty liver index [FLI], fibrosis-4 [FIB-4] index, and NAFLD fibrosis score [NFS]) were evaluated. The 743 subjects were classified into two groups: lean MetS (131 patients) and non-lean MetS (612 patients). The NAFLD prevalence in the non-lean group (62.6%) was higher than that in the lean group (31.3%). The age-adjusted odds ratio was 3.43. Advanced fibrosis was detected in 7.6% of lean patients and 10.8% of non-lean patients. FLI was not sensitive enough to detect NAFLD in the lean group at a high cutoff, but it performed acceptably at a low cutoff. FIB-4 performed better than NFS in determining advanced fibrosis. NAFLD was more common in non-lean than lean patients. Lean patients with MetS had a relatively higher risk of NAFLD than the general population. FLI and FIB-4 index performed acceptably in both groups.

## 1. Introduction

Non-alcoholic fatty liver disease (NAFLD) is characterized by significant lipid deposition in hepatocytes. NAFLD has a broad spectrum of liver damage, ranging from simple steatosis to steatohepatitis, advanced fibrosis, and cirrhosis [1]. It is one of the most common etiologies of chronic liver disease. NAFLD has become a public health concern due to its increasing prevalence, which has doubled over the last 20 years [2,3,4]. The global prevalence of NAFLD diagnosed by imaging has been estimated to be 25%. In Asia, its prevalence was estimated to be approximately 27% [3]. NAFLD increases the risks of hepatic-related diseases, such as cirrhosis and hepatocellular carcinoma, and extrahepatic consequences, like cardiovascular diseases and chronic kidney disease [5,6]. Additionally, many studies have reported increased mortality rates in patients with NAFLD [5,7].

NAFLD is associated with obesity; however, a recent meta-analysis showed a rising prevalence of NAFLD cases in the non-obese population. In fact, among the patients with NAFLD, almost one-fifth were non-obese [7,8,9,10,11]. The definition of lean NAFLD varies for different population groups according to variations in cut points for body mass index (BMI) and visceral obesity-waist circumference. In the Asian population, “lean NAFLD” is often used to describe a patient with NAFLD whose BMI is below 23 kg/m^2^ [12,13]. Many studies have shown that NAFLD and metabolic syndrome (MetS) have a bidirectional association. Not only are the features of MetS highly prevalent in patients with NAFLD, but the components of MetS also increase the risk of developing NAFLD [3,14]. These associations are experienced in lean and non-lean NAFLD groups. However, in the lean NAFLD group, the metabolic abnormalities are less profound, and the histological profile is less severe [9,10,15].

Even though the prevalence of lean NAFLD is increasing worldwide, data on lean NAFLD in the Thai population with MetS are still limited. This study focused on characterizing the demographic and clinical data and determining the prevalence of lean and non-lean NAFLD in patients with existing MetS. Furthermore, the study aimed to validate non-invasive scoring systems used to predict NAFLD and advanced fibrosis in the two groups.

## 2. Materials and Methods

### 2.1. Study Design and Ethics Approval

This was a cross-sectional study. Data on patients diagnosed with MetS at Siriraj Hospital, Bangkok, Thailand (a 2221-bed university hospital) [16] between January 2011 and December 2020 were collected by retrospective review of medical records. Before this research began, its protocol was approved by the Siriraj Institutional Review Board (approval number 102/2021).

### 2.2. Patient Selection

The inclusion criteria were adult patients ≥18 years of age who were diagnosed with MetS and had ultrasonography or transient elastography (FibroScan: Echosens, Paris, France) results. MetS was identified according to the guidelines of the National Cholesterol Education Program Adult Treatment Panel III (NCEP ATP III) 2005 [17,18] and the American Heart Association/National Heart Lung and Blood Institute (AHA/NHLBI) 2005 [19]. MetS was diagnosed by the presence of any 3 of the following 5 features:

- Waist circumference for Asian population ≥90 cm in men or ≥80 cm in women [18]
- Fasting blood sugar (FBS) ≥100 mg/dL or hypoglycemia agent usage
- High-density lipoprotein cholesterol (HDL-C) <40 mg/dL in men or <50 mg/dL in women
- Triglycerides (TG) ≥150 mg/dL or lipid-lowering drug usage
- Blood pressure ≥130/80 mmHg or antihypertensive drug usage [17,18,19]

The exclusion criterion was patients who had any secondary cause of hepatic steatosis. Examples of secondary causes are previous or current excessive alcohol intake (exceeding 30 g/day for men and 20 g/day for women), viral hepatitis, chronic liver disease, and drug-induced hepatitis [2,20,21]. Alcohol consumption behavior was investigated by taking routine medical history. Chronic viral hepatitis B and C which were prevalent in Thailand were identified by the hepatitis B surface antigen test and hepatitis C antibody test, respectively. Drug-induced hepatitis was investigated by reviewing patients’ medication lists. Patients with documented medical history of any chronic liver disease other than NAFLD were excluded.

### 2.3. Definition of Outcomes and Assessment of NAFLD and Significant and Advanced Fibrosis

In order to diagnose NAFLD, there must be:

- imaging or histological evidence of hepatic steatosis (defined as a lipid concentration > 5–10% of the liver weight), and
- a lack of secondary causes of hepatic fat accumulation (such as significant alcohol consumption, long-term use of a steatogenic medication, or monogenic hereditary disorders) [2,22].

In the present study, NAFLD was diagnosed by either a bright liver score ≥1 from conventional ultrasonography or a controlled attenuation parameter (CAP) greater than 275 decibels per meter (dB/m) from transient elastography [23]. Significant and advanced fibrosis were diagnosed when the liver stiffness measurement value from transient elastography was ≥7.0 kilopascals (kPa) and ≥8.0 kPa, respectively [23,24].

In order to evaluate the validities and performances of the three non-invasive scoring systems for predicting NAFLD and liver fibrosis in patients with MetS, we calculated the fatty liver index (FLI), Fibrosis-4 (FIB-4) index, and NAFLD fibrosis score (NFS) of the included patients. The FLI cutoffs used to indicate the presence of NAFLD were 30 and 60. The FIB-4 cutoffs to predict advanced fibrosis were 1.30 and 3.25. The NFS cutoffs were −1.455 and 0.676 [23,24]. The formulas of the three scoring systems are listed below:

$$\mathrm{FLI}=\frac{e^{0.953\times \ln\left(\mathrm{TG}\right)+0.139\times \mathrm{BMI}+0.718\times \ln\left(\mathrm{GGT}\right)+0.053\times \left(\mathrm{WC}\right)-15.745}}{1+e^{0.953\times \ln\left(\mathrm{TG}\right)+0.139\times \mathrm{BMI}+0.718\times \ln\left(\mathrm{GGT}\right)+0.053\times \left(\mathrm{WC}\right)-15.745}}\times 100$$

$$\text{FIB-4}=\frac{\mathrm{AGE}\times \mathrm{AST}}{\mathrm{platelet}\;\left(10^{9}\;\mathrm{per}\;\mathrm{liter}\right)\times {\mathrm{ALT}}^{1/2}}$$

$$\mathrm{NFS}=-1.675+0.037\times \mathrm{AGE}+0.094\times \mathrm{BMI}+1.13\times \mathrm{IFG}\;\mathrm{or}\;\mathrm{diabates}\;\mathrm{mellitus}\;\left(\mathrm{yes}=1,\;\mathrm{no}=0\right)\;+0.99\times \mathrm{AST}/\mathrm{ALT}\;\mathrm{ratio}-0.013\times \mathrm{platelet}\;\left(10^{9}\;\mathrm{per}\;\mathrm{liter}\right)-0.66\times \mathrm{albumin}$$

### 2.4. Sample Size Calculation

The sample size calculation was based on the prevalence of NAFLD in a cohort of Thai patients with MetS from a previous preliminary local data collection. The prevalence percentages of NAFLD among lean patients with MetS and non-lean patients with MetS were 51% and 77%, respectively. At alpha = 0.01 and power = 90%, the minimum sample size for each arm was 107 patients. However, a larger sample size is preferable, particularly to define the differences in risk factors for NAFLD between the two groups. Thus, we recruited all relevant patients with ultrasonography or transient elastography results during the study period.

### 2.5. Data Collection

The eligible patients were allocated to two groups: a lean group (BMI < 23 kg/m^2^) and a non-lean group (overweight patients with a BMI ≥ 23 kg/m^2^ but < 25 kg/m^2^; and obese patients with a BMI ≥ 25 kg/m^2^). Patient data were retrieved from electronic medical records. Age, sex, anthropomorphic data (body weight, height, waist circumference, hip circumference), date and results of ultrasonography or transient elastography, history of smoking, underlying diseases, and laboratory investigations (complete blood count, liver function test, FBS, hemoglobin A1c [HbA1c], and lipid profile) were collected. The blood test was performed either within 6 months before or after the assessment of hepatic steatosis and liver fibrosis. The results that had been measured closest to the date of assessment were chosen to use in the further analyses. We considered data were missing if there were no recorded data 6 months before and 6 months after the date of ultrasonography or transient elastography. All clinical and laboratory data were measured using standard techniques. The data were collected by internal medicine physicians with blinding to hepatic assessment results. Moreover, the scores of three non-invasive scoring systems were calculated after the data collection process was completed.

### 2.6. Statistical Analysis

Demographic data were analyzed and reported using descriptive statistics. Categorical variables were expressed as frequency and percentage. The mean and standard deviation or the median and interquartile range were used to summarize continuous variables.

Independent *t*-tests and Mann–Whitney U tests were used to compare normally distributed and non-normally-distributed continuous variables of the two groups, respectively. The proportions of the two groups were compared with Fisher’s exact test. Univariable and multivariable logistic regression models were applied to acquire odds ratios (ORs) and adjusted ORs of prevalence. A probability (*p*) value of less than 0.05 was considered statistically significant. All analyses were performed using Stata Statistical Software, release 15.1 (StataCorp LP, College Station, TX, USA).

## 3. Results

### 3.1. Demographic Data

The medical records of 969 patients with ultrasonography or transient elastography results were collected. Of these, 226 patients were excluded as they had secondary causes of hepatic disease. The remaining 743 patients met the inclusion and exclusion criteria and were enrolled in the study. Among the included patients, 131 (17.6%) were categorized as having lean MetS, while 612 (82.4%) were placed in the non-lean MetS group. Within the non-lean group, there were 161 overweight patients (26.3%) and 451 obese patients (73.7%).

The mean age of the study participants was 68.7 ± 11.5 years. Lean patients with MetS were older than non-lean patients (74.0 ± 11.1 versus 67.6 ± 11.3 years; *p* < 0.001). More than half were women (58.4%). The BMIs of patients with NAFLD were significantly higher than those without NAFLD in the lean and non-lean groups. However, the waist circumference of patients with NAFLD was significantly higher than that of patients without NAFLD in the non-lean group.

Interestingly, the mean age of the non-NAFLD patients was higher than that of the patients with NAFLD (70.1 ± 10.5 versus 67.6 ± 12.1 years; *p* = 0.003). In the NAFLD group, more than half of the patients had diabetes mellitus, and they were in both the lean and non-lean groups. Most of the patients had hypertension and dyslipidemia. Prevalence numbers of diabetes mellitus among patients with NAFLD were significantly higher than those without NAFLD in both groups. There were also significant differences in the ALT, GGT, TG, and HDL-C levels of patients with and without NAFLD in the lean and non-lean groups. Details are presented in Table 1.

### 3.2. Clinical Outcomes and Prevalence of NAFLD and Liver Fibrosis

The overall prevalence of NAFLD in patients with MetS was 57.1% (424 of 743 patients; 95% confidence interval [CI], 53.4–60.7%). Two-thirds of the non-lean patients had NAFLD, whereas only one-third of the lean patients had NAFLD (62.6% [95% CI, 58.6–66.4%] versus 31.3% [95% CI, 23.5–40.0%]; OR = 3.67 [95% CI, 2.45–5.50]; *p* < 0.001). When adjusted by age, the OR of the NAFLD prevalence of the non-lean versus lean patients was 3.43 (95% CI, 2.27–5.17; *p* < 0.001). The FLI was significantly higher for patients with NAFLD. However, there were no statistical differences in the values of FIB-4 index and NFS of both groups.

In lean patients with MetS, the severity of liver fibrosis was lower than for non-lean patients with MetS. The prevalence of NAFLD with significant fibrosis was 7.6% (95% CI, 3.7–13.6%) and 13.7% (95% CI, 11.1–16.7%) in lean and non-lean patients with MetS, respectively (OR = 1.92; 95% CI, 0.97–3.82; *p* = 0.061). The age-adjusted OR was 2.13 (95% CI, 1.06–4.28; *p* = 0.034). Advanced fibrosis was detected in 7.6% (95% CI, 3.7–13.6%) and 10.8% (95% CI, 8.4–13.5%) of lean and non-lean patients with MetS, respectively (OR = 1.46; 95% CI, 0.73–2.93; *p* = 0.283; and age-adjusted OR = 1.76; 95% CI, 0.86–3.58; *p* = 0.120). The AST, ALT, and GGT levels were higher for patients with advanced fibrosis, but platelets and LDL-C were lower for the advanced fibrosis group, in both lean and non-lean patients. Having diabetes mellitus was associated with advanced fibrosis, but the statistical significance was observed only in non-lean patients (Table 2).

There were significant differences in the clinical parameters of waist circumference, FBS, HbA1c, TG, and HDL-C for patients with and without advanced fibrosis, but only for those in the non-lean group. Interestingly, among lean patients with MetS, those with advanced fibrosis were older, had higher ALP, and had lower albumin than those without significant fibrosis. Regarding the prediction of fibrosis, the FIB-4 index and NFS scores of patients with advanced fibrosis were significantly higher than those without advanced fibrosis. Details are shown in Table 2.

Among patients with NAFLD (*n* = 424), BMI, waist circumference, hip circumference, and ALT were significantly higher for those in the non-lean NAFLD group than for the lean NAFLD group. In contrast, age and HbA1c were lower in the non-lean NAFLD group. The proportions of lean and non-lean patients with NAFLD who also had underlying diseases were similar. The data are listed in Table 3.

### 3.3. Performances of Scoring Systems for Predicting NAFLD and Liver Fibrosis

**Figure 1:**
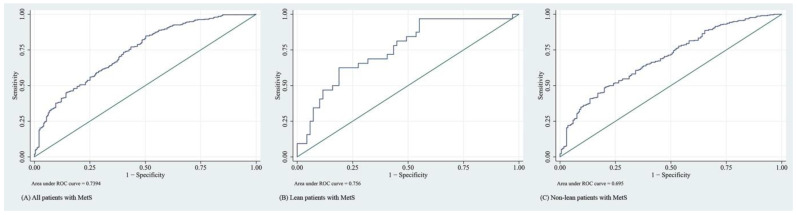
Receiver operating characteristic curves of the FLI for predicting NAFLD of (**A**) all patients with MetS, (**B**) lean patients with MetS, and (**C**) non-lean patients with MetS.

The overall performance of FLI was acceptable when used to predict NAFLD (area under the receiver operating characteristic [AUROC] = 0.70–0.76). The receiver operating characteristic plots are illustrated in Figure 1. As shown in Table 4, we determined the accuracy of FLI for predicting the presence of NAFLD at two cutoffs: FLI ≥ 30 and FLI > 60. At both cutoffs, the sensitivities of the scoring system in predicting NAFLD were lower, and its specificities were higher for non-lean patients than lean patients. However, the scoring system did not perform well in the subgroup of lean patients with MetS if the cutoff of 60 was applied (AUROC = 0.55; 95% CI, 0.50–0.60).

**Figure 2:**
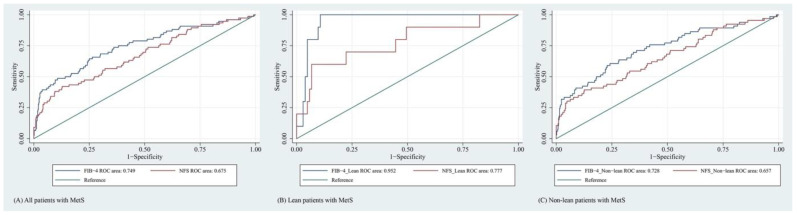
Receiver operating characteristic curves of FIB-4 and NFS for predicting NAFLD with advanced fibrosis of (**A**) all patients with MetS, (**B**) lean patients with MetS, and (**C**) non-lean patients with MetS.

According to the results, the FIB-4 index performed significantly better than NFS in predicting NAFLD with advanced fibrosis among patients with MetS (AUROC = 0.749 [95% CI, 0.683–0.814] versus 0.675 [95% CI, 0.606–0.744], respectively; *p* = 0.001). When the lean and non-lean groups were compared, a higher AUROC was observed with the lean group. At its lower cutoff, FIB-4 provided high AUROC, with an AUROC value of above 0.70 in the lean subgroup. Unfortunately, its PPV was very poor, barely above 10.0%. The finding indicated that the lower cutoff for FIB-4 would not be appropriate for the lean group as it was prone to false positives. This problem was also seen when determining the outcome using NFS at its lower cutoff. Details are presented in Figure 2 and Table 5.

## 4. Discussion

The NAFLD prevalence percentages diagnosed by ultrasonography or transient elastography of the overall cohort (57.1%), lean group (31.3%), and non-lean group (62.6%) were higher than the values of previous studies. The higher rates resulted from the cohort of the present investigation when only including patients with MetS. Earlier research showed that patients diagnosed with MetS had a greater risk of developing NAFLD than those without MetS (OR = 1.72; *p* = 0.015) [20,25,26]. Moreover, a large population-based study in the United States on the association between NAFLD and metabolic abnormalities reported a significantly higher prevalence of NAFLD among patients with MetS than those without MetS (adjusted OR = 11.5; 95% CI, 8.9–14.7) [25]. In addition, Goyal et al. [27] and Shaikh et al. [28] observed similar results, with prevalence percentages of NAFLD among patients with MetS of 73% and 43%, respectively.

Furthermore, the current work discovered that the prevalence of NAFLD among non-lean patients with MetS was significantly higher than that of lean patients with MetS (62.6% [95% CI, 58.6–66.4%] versus 31.3% [95% CI, 23.5–40.0%], respectively). There is evidence that the risk of NAFLD is associated with age [29]. In our study, the lean group was older than the non-lean group. However, adjusted by age, the OR comparing the prevalence of NAFLD in the lean and non-lean groups still showed a significant value of 3.43 (95% CI, 2.27–5.17). Compared with other studies, our proportion of NAFLD in both lean and non-lean patients with MetS was remarkably higher. Younossi et al. found that among the 11 613 eligible participants in the National Health and Nutrition Examination Survey III (NHANES III) in the United States, the prevalence percentages of NAFLD were 7.39% and 27.75% for the lean and overweight/obese populations, respectively [30]. A recent systematic review and meta-analysis gathered 33 observational studies with205,307 individuals from 14 countries. It reported that the prevalence percentages of NAFLD from 2010 onwards were 11.8% in lean subjects and 51.3% in non-lean subjects. The same study found that the prevalence of lean NAFLD in Asia was 3.8% to 5.5%, varying by country [31]. Therefore, it is essential to note that our population consisted of high-risk patients, which might explain the higher prevalence in our lean and non-lean groups.

Surprisingly, we found that patients not diagnosed with NAFLD were older than those with NAFLD. This conflicts with most other evidence [29]. However, when our patients were classified into lean and non-lean groups, only the non-lean group showed that younger patients were associated with a diagnosis of NAFLD. This result might establish that age is not a predictor of NAFLD among lean patients with MetS. On the other hand, we found that age was a predictor of advanced fibrosis only in the subgroup of lean patients with MetS. Men have a higher prevalence and severity of NAFLD throughout reproductive years than women. However, after menopause, NAFLD occurs at a higher rate in women, suggesting estrogen is a protective factor in premenopausal women [32]. Although most of the women in our cohort were in the menopausal stage, we did not observe an association between sex and the risk of NAFLD or advanced fibrosis in lean or non-lean patients with MetS.

The association between diabetes mellitus and NAFLD-related outcomes was observed in the present study. The proportion of patients with diabetes mellitus was significantly higher in the groups of patients with NAFLD, both lean and non-lean. A previous study by Masarone et al. [33] found that NAFLD is clinical signs of type 2 diabetes mellitus. Moreover, non-alcoholic steatohepatitis was present in almost all patients with type 2 diabetes mellitus and more than half of patients with MetS were diagnosed with non-alcoholic steatohepatitis which could further progress to advanced fibrosis. Several recent studies have investigated the factors associated with NAFLD and MetS, diabetes mellitus, or cardiovascular diseases. They discovered that various genetic factors, including patatin-like phospholipase domain-containing protein 3 (PNPLA3), and transmembrane 6 superfamily member 2 (TM6SF2), play crucial roles in NAFLD development and progression of its consequences [34,35]. Additionally, it had been proven that insulin resistance increases hepatic lipogenesis and decreases a suppression of lipolysis in the adipose tissue, and results in an excess of fat accumulates in the liver [34,35,36,37,38].

The findings of this study support the recommendation that lifestyle modification and weight reduction should be employed as central treatment strategies for lean patients with NAFLD [7]. We found that non-lean and lean patients with NAFLD had significantly higher BMIs than those without the disease. Our findings correspond to those of other studies that reported that patients with NAFLD had a higher average BMI and higher odds of central obesity than the general population [31,39]. The recommendation of lifestyle modification and weight reduction is mandatory in patients with NAFLD as both lean and non-lean patients are at an increased risk of death [7].

Regarding the liver stiffness outcome, lean subjects tended to have a lower prevalence of advanced fibrosis. However, this finding did not reach statistical significance. The non-significant outcome might be due to the small proportion of patients with the outcome and the confounding effects of unknown factors. Compared with non-lean patients with advanced fibrosis, lean patients were older and had higher ALP. Previous studies showed varying results. Young et al. compared the profiles of lean and obese patients with NAFLD. They found no difference in the mean ages of the groups, but the lean patients with NAFLD had less severe liver fibrosis than the obese patients with NAFLD [10]. Another recent study in China found that patients with lean NAFLD were more likely to be older and male and have lower liver stiffness values than patients with non-lean NAFLD [40].

Moving on to the laboratory parameters, we found that elevated serum ALT levels were related to hepatic steatosis and fibrosis in patients with MetS. Moreover, serum ALT was generally higher in non-lean patients than lean patients. Earlier research showed that a rising ALT level was strongly related to a high waist circumference and a high BMI. Furthermore, an increased ALT level was related to steatohepatitis and intense fibrosis [41]. Regarding diabetic status, our participants appeared to be well controlled, except for lean patients with NAFLD (with and without advanced fibrosis). This finding might be because the non-lean group comprised a higher proportion of younger patients, who may be more capable of controlling their blood sugar levels.

Concerning the subgroup of patients with NAFLD, approximately 10% were lean. This percentage is consistent with the prevalence of lean NAFLD in Asian countries [7]. However, it is lower than the values reported in a meta-analysis by Alam et al. [42] and other studies [11,43]. One explanation is that our study was conducted exclusively on patients with MetS. Similar to other studies, we found those lean patients with MetS were significantly older and tended to have higher diabetes-related risks (such as higher levels of FBS and HbA1c) than non-lean patients with MetS [11,42].

The FLI, a widely used non-invasive scoring system for predicting NAFLD, performed acceptably in our MetS cohort. The predictive performance of this scoring system correlated with a previous study that diagnosed NAFLD in patients with MetS solely by ultrasonography [44]. The FLI scoring system had poor sensitivity for detecting NAFLD in lean patients at a high cutoff. This result corresponds with the work of Hsu et al., who enrolled 4000 lean patients with NAFLD (BMI < 24 kg/m^2^). However, they discovered that FLI was an appropriate scoring system for predicting NAFLD among lean individuals at a proper cutoff (FLI ≥ 15, which provided the highest discriminative ability) [45].

Two predictive scoring systems for advanced fibrosis were validated in our MetS cohort. The results indicated that the FIB-4 index performed significantly better than NFS in predicting NAFLD with advanced fibrosis. NFS did not perform well in our whole cohort. However, it appeared to be a more acceptable and superior screening tool in lean patients than non-lean patients in terms of its AUROC value. Even if the FIB-4 index seemed to be a promising non-invasive test for advanced fibrosis in lean patients with MetS, it struggled to identify true-positive patients in our cohort. Previous evidence did mention that the false-positive rates of FIB-4 and NFS increased when scores were applied in patients aged ≥ 65 years [23].

There are several limitations to our study. First, we did not use liver biopsies to diagnose NAFLD and determine the severity of liver fibrosis. Although liver biopsy is the gold standard for diagnosing NAFLD, the procedure is invasive and frequently associated with distress and discomfort. For a large-scale study, such as the current investigation, ultrasonography and transient elastography are preferred because they are more practicable than a biopsy [20]. The most common imaging method used to diagnose hepatic steatosis is conventional ultrasonography since it is widely available, innocuous, inexpensive, and well established [20]. In a large meta-analysis, ultrasonography’s pooled sensitivity and specificity for detecting hepatic steatosis were 85% and 94%, respectively [46]. The sensitivity and PPV of hepatic steatosis diagnosis by using CAP value were over 90% [23]. The mean sensitivity and specificity of liver stiffness evaluation from transient elastography were more than 70%, with an AUROC curve value of 0.82 [47,48]. Second, subgroup analyses of the overweight and obese subgroups were not carried out. As well, no analysis of patients classified with visceral obesity (via waist circumference) was performed. We believed that BMI might be a more reliable measurement method to identify lean individuals than waist circumference. Third, despite all available patients with MetS between 2011 and 2020 being recruited, the number of patients with advanced fibrosis was still low. Consequently, there was insufficient power to distinguish the prevalence of advanced fibrosis between lean and non-lean patients with MetS. Fourth, the average age gaps between lean and non-lean patients with MetS as well as those with and without NAFLD were quite large. Even if the outcomes, regarding difference of the NAFLD prevalence and severity of liver fibrosis, were addressed by multivariable logistic regression (adjusted for age), future researches with different study design that include younger participants are required to confirm our findings. Finally, this study was a single-center study that included only Thai patients to the cohort. Thus, the results might not be able to be applied to other ethnic groups. Moreover, the older cohort might also impact the accuracy of the predictive performances of the three non-invasive scoring systems. Therefore, the generalizability of the outcomes should be considered.

Our study was the first to focus exclusively on the distinctions between lean and non-lean Asian patients with MetS. A higher prevalence of NAFLD was observed in patients with MetS in both the non-lean and lean groups. Even though the prevalence of NAFLD was significantly higher in the non-lean group, patients with lean NAFLD still had a higher average BMI than lean patients without the disease. Some clinical parameters and risk factors related to NAFLD differed from those reported by other studies that explored the outcomes of interest in populations of other ethnicities. Additionally, we provided external validations of three standard non-invasive scoring systems related to NAFLD and its consequences in Asian populations with MetS. We also examined and illustrated the differences in the predictive performances for the lean and non-lean groups. Given that a liver biopsy is not recommended for screening individuals without specific conditions, our study compared non-invasive scoring systems with ultrasonography and transient elastography results [40]. This approach provided valuable information that can be applied to general practice.

## 5. Conclusions

NAFLD was significantly more common in non-lean patients with MetS. Approximately one-third of lean patients with MetS and two-thirds of non-lean patients with MetS were diagnosed with NAFLD. Compared with the general population, lean patients with MetS had a relatively higher risk of NAFLD. Both non-lean and lean patients with NAFLD should be introduced to proper treatment. The validities of FLI for predicting NAFLD and of FIB-4 for predicting NAFLD with advanced fibrosis in the overall cohort and the subgroups of lean and non-lean patients were acceptable. However, the validity of NFS should be considered in patients with MetS.

## Figures and Tables

**Table 1:** Demographic data of patients with and without NAFLD.

Demographic Data	Lean (*n* = 131)			Non-Lean (*n* = 612)		
	Non-NAFLD (*n* = 90, 68.7%)	NAFLD (*n* = 41, 31.3%)	*p*	Non-NAFLD (*n* = 229, 37.4%)	NAFLD (*n* = 383, 62.6%)	*p*
Age (year)	73.5 ± 11.4	75.0 ± 10.7	0.461	68.8 ± 9.9	66.8 ± 11.9	0.033
Gender: male (*n*, %)	31 (34.4)	16 (39.0)	0.695	90 (39.3)	172 (44.9)	0.178
BMI (kg/m^2^)	20.9 ± 1.6	21.6 ± 1.5	0.019	26.7 ± 2.9	28.2 ± 4.1	<0.001
Waist circumference (cm)	79.4 ± 9.8	83.0 ± 7.5	0.056	91.7 ± 8.6	95.6 ± 9.5	<0.001
Hip circumference (cm)	89.2 ± 4.0	91.0 ± 4.7	0.101	100.3 ± 7.4	100.7 ± 7.1	0.667
Smoker/ex-smoker (*n*, %)	9 (10.0)	4 (9.8)	1.000	124 (10.5)	27 (7.1)	0.173
DM (*n*, %)	32 (35.6)	25 (61.0)	0.008	80 (34.9)	206 (53.8)	<0.001
HT (*n*, %)	70 (77.8)	37 (90.2)	0.096	200 (87.3)	339 (88.5)	0.700
DLP (*n*, %)	87 (96.7)	41 (100.0)	0.552	223 (97.4)	369 (96.3)	0.640
AST median (IQR) (mg/dL)	21.0 (17.0, 25.0)	22.0 (18.0, 27.0)	0.329	21.0 (18.0, 26.0)	24.0 (20.0, 31.0)	<0.001
ALT median (IQR) (mg/dL)	17.0 (13.0, 20.0)	19.5 (15.0, 30.5)	0.014	19.0 (15.0, 25.0)	26.0 (19.0, 37.0)	<0.001
ALP median (IQR) (mg/dL)	70.0 (53.0, 85.0)	70.0 (62.0, 94.0)	0.081	69.0 (59.0, 84.0)	68.0 (58.0, 84.0)	0.782
GGT median (IQR) (mg/dL)	20.0 (15.0, 30.0)	37.0 (23.0, 60.5)	<0.001	25.0 (21.0, 36.0)	34.0 (23.0, 58.5)	<0.001
Albumin (g/dL)	4.4 ± 0.3	4.4 ± 0.4	0.868	4.4 ± 0.3	4.4 ± 0.3	0.020
FBS (mg/dL)	114.1 ± 35.4	130.7 ± 61.4	0.053	114.6 ± 39.8	119.7 ± 33.0	0.087
HbA1c (%)	6.1 ± 0.9	6.8 ± 1.4	<0.001	6.3 ± 1.1	6.4 ± 0.9	0.052
Total cholesterol (mg/dL)	180.0 ± 37.9	171.7 ± 32.3	0.229	173.5 ± 38.7	175.6 ± 36.4	0.489
TG median (IQR) (mg/dL)	89.0 (69.0, 125.0)	115.0 (98.0, 167.0)	0.003	105.0 (78.0, 147.0)	131.0 (97.0, 173.0)	<0.001
HDL-C (mg/dL)	62.4 ± 16.6	54.0 ± 16.1	0.008	55.2 ± 14.0	51.6 ± 12.4	0.001
LDL-C (mg/dL)	98.1 ± 33.1	90.2 ± 31.4	0.204	95.7 ± 32.0	94.6 ± 31.9	0.671
Significant fibrosis (*n*, %)	0 (0.0)	10 (24.4)	-	0 (0.0)	84 (21.9)	-
Advanced fibrosis (*n*, %)	0 (0.0)	10 (24.4)	-	0 (0.0)	66 (17.2)	-
FLI	10.9 (6.6, 20.3)	24.8 (12.8, 39.7)	<0.001	42.1 (25.3, 58.1)	59.0 (40.1, 78.8)	<0.001
FIB-4	1.469 (1.053, 2.098)	1.470 (1.018, 2.480)	0.779	1.337 (1.023, 1.797)	1.204 (0.928, 1.702)	0.100
NFS	−0.842 (−1.962, 0.091)	−0.913 (−2.266, −0.054)	0.961	−0.720 (−1.468, −0.025)	−0.872 (−1.702, −0.059)	0.354

ALP, alkaline phosphatase; ALT, alanine aminotransferase; AST, aspartate aminotransferase; BMI, body mass index; DLP, dyslipidemia; DM, diabetes mellitus; FBS, fasting blood sugar; FIB-4, fibrosis-4; FLI, fatty liver index; GGT, gamma-glutamyl transferase; HbA1c, hemoglobin A1c; HDL-C, high-density lipoprotein cholesterol; HT, hypertension; LDL-C, low-density lipoprotein cholesterol; NAFLD, non-alcoholic fatty liver disease; NFS, NAFLD fibrosis score; TG, triglycerides.

**Table 2:** Demographic data of patients with and without advanced fibrosis.

Demographic Data	Lean (*n* = 131)			Non-Lean (*n* = 612)		
	Non-Advanced Fibrosis (*n* = 121, 92.4%)	Advanced Fibrosis (*n* = 10, 7.6%)	*p*	Non-Advanced Fibrosis (*n* = 546, 89.2%)	Advanced Fibrosis (*n* = 66, 10.8%)	*p*
Age (year)	73.2 ± 10.8	83.4 ± 11.6	0.005	67.3 ± 10.9	69.7 ± 13.7	0.098
Gender: male (*n*, %)	43 (35.5)	4 (40.0)	0.754	230 (42.1)	32 (48.5)	0.357
BMI (kg/m^2^)	21.1 ± 1.5	21.0 ± 2.1	0.796	27.5 ± 3.6	28.8 ± 4.2	0.008
Waist circumference (cm)	80.3 ± 9.1	85.0 ± 12.4	0.275	93.7 ± 9.2	98.6 ± 10.0	<0.001
Hip circumference (cm)	89.9 ± 4.4	93.5 ± 3.5	0.247	100.6 ± 7.2	101.0 ± 7.3	0.763
Smoker/ex-smoker (*n*, %)	12 (9.9)	1 (10.0)	1	44 (8.1)	7 (10.6)	0.479
DM (*n*, %)	50 (41.3)	7 (70.0)	0.102	235 (43.0)	51 (77.2)	<0.001
HT (*n*, %)	97 (80.2)	10 (100.0)	0.207	478 (87.6)	61 (92.4)	0.316
DLP (*n*, %)	118 (97.5)	10 (100.0)	1	527 (96.5)	65 (98.5)	0.712
AST median (IQR) (mg/dL)	21.0 (17.0, 25.0)	64.0 (26.0, 89.0)	<0.001	22.0 (18.0, 27.0)	35.0 (28.0, 44.0)	<0.001
ALT median (IQR) (mg/dL)	17.0 (13.0, 20.0)	43.5 (21.0, 80.0)	0.001	22.0 (16.0, 31.0)	34.5 (25.0, 53.0)	<0.001
ALP median (IQR) (mg/dL)	69.5 (55.0, 84.0)	109.5 (75.0, 165.0)	0.002	68.0 (58.0, 84.0)	71.5 (62.0, 92.0)	0.069
GGT median (IQR) (mg/dL)	22.0 (17.0, 35.0)	94.5 (43.5, 405)	<0.001	29.0 (21.0, 46.0)	57.0 (35.0, 91.5)	<0.001
Albumin (g/dL)	4.4 ± 0.3	4.1 ± 0.4	0.007	4.4 ± 0.3	4.4 ± 0.4	0.118
Platelet (×10^3^/mm^3^)	264.3 ± 80.3	197.4 ± 47.8	0.011	264.6 ± 64.8	221.9 ± 79.6	<0.001
FBS (mg)	118.9 ± 47.0	123.9 ± 25.1	0.742	116.5 ± 35.2	128.6 ± 38.6	0.010
HbA1c (%)	6.3 ± 1.1	7.0 ± 1.5	0.061	6.3 ± 0.9	6.8 ± 1.3	<0.001
Total cholesterol (mg/dL)	179.1 ± 36.5	157.5 ± 29.6	0.071	175.6 ± 37.6	168.1 ± 34.3	0.119
TG median (IQR) (mg/dL)	102.0 (73.0, 133.5)	114.5 (101.0, 125.0)	0.450	119.0 (86.0, 159.0)	137.5 (112.0, 180.0)	0.003
HDL-C (mg/dL)	59.9 ± 16.4	58.7 ± 22.0	0.836	53.4 ± 13.2	49.2 ± 11.8	0.014
LDL-C (mg/dL)	97.2 ± 32.2	75.8 ± 33.1	0.046	96.1 ± 32.0	85.6 ± 29.5	0.012
FIB-4	1.420 (1.038, 2.040)	3.219 (2.789, 4.023)	<0.001	1.218 (0.945, 1.640)	1.812 (1.341, 3.038)	<0.001
NFS	−1.043 (−2.094, −0.086)	0.571 (−0.735, 0.726)	0.004	−0.879 (−1.696, −0.104)	−0.247 (−1.224, 1.131)	<0.001

ALP, alkaline phosphatase; ALT, alanine aminotransferase; AST, aspartate aminotransferase; BMI, body mass index; DLP, dyslipidemia; DM, diabetes mellitus; FBS, fasting blood sugar; FIB-4, fibrosis-4; GGT, gamma-glutamyl transferase; HbA1c, hemoglobin A1c; HDL-C, high-density lipoprotein cholesterol; HT, hypertension; LDL-C, low-density lipoprotein cholesterol; NFS, NAFLD fibrosis score; TG, triglycerides.

**Table 3:** Comparison of lean patients with NAFLD and non-lean patients with NAFLD.

NAFLD (*n* = 424)	Lean with NAFLD (*n* = 41, 9.7%)	Non-Lean with NAFLD (*n* = 383, 90.3%)	*p*
Age (year)	75.0 ± 10.7	66.8 ± 11.9	<0.001
Gender: male (*n*, %)	16 (39.0)	172 (44.9)	0.512
BMI (kg/m^2^)	21.6 ± 1.5	28.2 ± 4.1	<0.001
Waist circumference (cm)	83.0 ± 7.5	95.6 ± 9.5	<0.001
Hip circumference (cm)	91.0 ± 4.7	100.7 ± 7.1	<0.001
Smoker/ex-smoker (*n*, %)	4 (9.8)	27 (7.1)	0.525
DM (*n*, %)	25 (61.0)	206 (53.8)	0.413
HT (*n*, %)	37 (90.2)	339 (88.5)	1.000
DLP (*n*, %)	41 (100.0)	369 (96.3)	0.379
AST median (IQR) (mg/dL)	22.0 (18.0, 27.0)	24.0 (20.0, 31.0)	0.164
ALT median (IQR) (mg/dL)	19.5 (15.0, 30.5)	26.0 (19.0, 37.0)	0.009
ALP median (IQR) (mg/dL)	70.0 (62.0, 94.0)	68.0 (58.0, 84.0)	0.105
GGT median (IQR) (mg/dL)	37.0 (23.0, 60.5)	34.0 (23.0, 58.5)	0.673
Albumin (g/dL)	4.4 ± 0.4	4.4 ± 0.3	0.300
FBS	130.7 ± 61.4	119.7 ± 33.0	0.069
HbA1c (%)	6.8 ± 1.4	6.4 ± 0.9	0.013
Total cholesterol (mg/dL)	171.7 ± 32.3	175.6 ± 36.4	0.511
TG median (IQR) (mg/dL)	115.0 (98.0, 167.0)	131.0 (97.0, 173.0)	0.340
HDL-C (mg/dL)	54.0 ± 16.1	51.6 ± 12.4	0.256
LDL-C (mg/dL)	90.2 ± 31.4	94.6 ± 31.9	0.406
Significant fibrosis (*n*, %)	10 (24.4)	84 (21.9)	0.695
Advanced fibrosis (*n*, %)	10 (24.4)	66 (17.2)	0.283

ALP, alkaline phosphatase; ALT, alanine aminotransferase; AST, aspartate aminotransferase; BMI, body mass index; DLP, dyslipidemia; DM, diabetes mellitus; FBS, fasting blood sugar; GGT, gamma-glutamyl transferase; HbA1c, hemoglobin A1c; HDL-C, high-density lipoprotein cholesterol; HT, hypertension; LDL-C, low-density lipoprotein cholesterol; NAFLD, non-alcoholic fatty liver disease; TG, triglycerides.

**Table 4:** Performance of FLI in predicting NAFLD among patients with MetS.

Fatty Liver Index (FLI)	*n*	AUROC	Sensitivity (%)	Specificity (%)	PPV (%)	NPV (%)	LR+	LR−
FLI ≥ 30								
All	589	0.67 (0.63, 0.71)	84.0 (79.7, 87.7)	49.8 (43.3, 56.3)	71.0 (66.4, 75.3)	68.0 (60.5, 74.8)	1.67 (1.46, 1.91)	0.32 (0.24, 0.42)
Lean	101	0.67 (0.57, 0.77)	46.9 (29.1, 65.3)	87.0 (76.7, 93.9)	62.5 (40.6, 81.2)	77.9 (67.0, 86.6)	3.59 (1.76, 7.33)	0.61 (0.44, 0.86)
Non-lean	488	0.61 (0.57, 0.65)	87.7 (83.6, 91.1)	34.7 (27.6, 42.4)	71.5 (66.8, 76.0)	60.2 (49.8, 70.0)	1.34 (1.20, 1.51)	0.35 (0.25, 0.51)
FLI > 60								
All	589	0.65 (0.62, 0.69)	45.4 (40.1, 50.8)	85.4 (80.2, 89.6)	82.0 (75.8, 87.1)	51.6 (46.6, 56.7)	3.10 (2.24, 4.30)	0.64 (0.57, 0.71)
Lean	101	0.55 (0.50, 0.60)	9.4 (2.0, 25.0)	100 (94.8, 100.0)	100 (29.2, 100.0)	70.4 (60.3, 79.2)	NA	0.91 (0.81, 1.01)
Non-lean	488	0.64 (0.60, 0.68)	49.1 (43.4, 54.7)	79.4 (72.5, 85.2)	81.7 (75.4, 86.9)	45.5 (39.7, 51.3)	2.38 (1.74, 3.27)	0.64 (0.56, 073)

AUROC, area under the receiver operating characteristic curve; LR, likelihood ratio; MetS, metabolic syndrome; NA, not applicable; NAFLD, non-alcoholic fatty liver disease; NPV, negative predictive value; PPV, positive predictive value.

**Table 5:** Performance of FIB-4 and NFS in predicting NAFLD with advanced fibrosis among patients with MetS.

Scores	*n*	AUROC	Sensitivity (%)	Specificity (%)	PPV (%)	NPV (%)	LR+	LR−
Fibrosis-4 (FIB-4)								
FIB-4 ≥ 1.3								
All	726	0.67 (0.62, 0.72)	78.9 (68.1, 87.5)	54.8 (50.9, 58.6)	16.9 (13.2, 21.3)	95.7 (93.1, 97.5)	1.75 (1.51, 2.02)	0.38 (0.25, 0.60)
Lean	127	0.71 (0.66, 0.75)	100.0 (69.2, 100.0)	41.9 (32.8, 51.4)	12.8 (6.3, 22.3)	100.0 (92.7, 100.0)	1.72 (1.48, 2.01)	NA
Non-lean	599	0.67 (0.61, 0.72)	75.8 (63.6, 85.5)	57.6 (53.3, 61.8)	18.1 (13.8, 23.2)	95.0 (92.1, 97.1)	1.79 (1.51, 2.11)	0.42 (0.27, 0.65)
FIB-4 ≥ 3.25								
All	726	0.60 (0.56, 0.65)	22.4 (13.6, 33.4)	98.2 (96.8, 99.0)	58.6 (38.9, 76.5)	91.5 (89.2, 93.5)	12.12 (6.02, 24.39)	0.79 (0.70, 0.89)
Lean	127	0.73 (0.56, 0.89)	50.0 (18.7, 81.3)	95.7 (90.3, 98.6)	50.0 (18.7, 81.3)	95.7 (90.3, 98.6)	11.70 (4.06, 33.71)	0.52 (0.28, 0.97)
Non-lean	599	0.58 (0.54, 0.63)	18.2 (9.8, 29.6)	98.7 (97.3, 99.5)	63.2 (38.4, 83.7)	90.7 (88.0, 92.9)	13.84 (5.65, 33.93)	0.83 (0.74, 0.93)
NAFLD fibrosis score (NFS)								
NFS ≥ −1.455								
All	655	0.59 (0.54, 0.63)	84.2 (74.0, 91.6)	32.8 (29.0, 36.8)	14.1 (11.1, 17.7)	94.1 (89.9, 96.9)	1.25 (1.12, 1.40)	0.48 (0.28, 0.82)
Lean	113	0.65 (0.54, 0.76)	90.0 (55.5, 99.7)	40.8 (31.2, 50.9)	12.9 (6.1, 23.0)	97.7 (87.7, 99.9)	1.52 (1.17, 1.97)	0.25 (0.04, 1.60)
Non-lean	542	0.57 (0.52, 0.62)	83.3 (72.1, 91.4)	31.1 (27.0, 35.5)	14.4 (11.0, 18.3)	93.1 (88.0, 96.5)	1.21 (1.07, 1.37)	0.54 (0.31, 0.93)
NFS ≥ 0.676								
All	655	0.63 (0.58, 0.69)	34.2 (23.7, 46.0)	92.4 (89.9, 94.4)	37.1 (25.9, 49.5)	91.5 (88.9, 93.6)	4.50 (2.95, 6.86)	0.71 (0.60, 0.84)
Lean	113	0.67 (0.51, 0.83)	40.0 (12.2, 73.8)	94.2 (87.8, 97.8)	40.0 (12.2, 73.8)	94.2 (87.8, 97.8)	6.87 (2.32, 20.34)	0.64 (0.38, 1.06)
Non-lean	542	0.63 (0.57, 0.69)	33.3 (22.2, 46.0)	92.0 (89.2, 94.3)	36.7 (24.6, 50.1)	90.9 (87.9, 93.3)	4.18 (2.64, 6.60)	0.72 (0.61, 0.86)

AUROC, area under the receiver operating characteristic curve; LR, likelihood ratio; MetS, metabolic syndrome; NA, not applicable; NAFLD, non-alcoholic fatty liver disease; NPV, negative predictive value; PPV, positive predictive value.

## Data Availability

The data presented in this study are available on request from the corresponding author. The data are not publicly available due to privacy and ethical reasons.

## Abbreviations

ALP: alkaline phosphatase; ALT, alanine aminotransferase; AST, aspartate aminotransferase; AUROC, area under the receiver operating characteristic; BMI, body mass index; CAP, controlled attenuation parameter; CI, confidence interval; DLP, dyslipidemia; DM, diabetes mellitus; FBS, fasting blood sugar; FIB-4, fibrosis-4; FLI, fatty liver index; GGT, gamma-glutamyl transferase; HbA1c, hemoglobin A1c; HDL-C, high-density lipoprotein cholesterol; HT, hypertension; kPa: kilopascals; LDL-C, low-density lipoprotein cholesterol; LR, likelihood ratio; MetS, metabolic syndrome; NAFLD, non-alcoholic fatty liver disease; NFS, NAFLD fibrosis score; NPV, negative predictive value; OR, odds ratio; PPV, positive predictive value; TC, total cholesterol; TG, triglycerides.

## References

[1] Abd El-Kader S.M., El-Den Ashmawy E.M. (2015). Non-alcoholic fatty liver disease: The diagnosis and management. World J. Hepatol..

[2] Chalasani N., Younossi Z., Lavine J.E., Charlton M., Cusi K., Rinella M., Harrison S.A., Brunt E.M., Sanyal A.J. (2018). The diagnosis and management of nonalcoholic fatty liver disease: Practice guidance from the American Association for the Study of Liver Diseases. Hepatology.

[3] Younossi Z.M., Koenig A.B., Abdelatif D., Fazel Y., Henry L., Wymer M. (2016). Global epidemiology of nonalcoholic fatty liver disease-Meta-analytic assessment of prevalence, incidence, and outcomes. Hepatology.

[4] Vernon G., Baranova A., Younossi Z.M. (2011). Systematic review: The epidemiology and natural history of non-alcoholic fatty liver disease and non-alcoholic steatohepatitis in adults. Aliment. Pharmacol. Ther..

[5] Zarghamravanbakhsh P., Frenkel M., Poretsky L. (2021). Metabolic causes and consequences of nonalcoholic fatty liver disease (NAFLD). Metabol. Open.

[6] Kim M.N., Han K., Yoo J., Ha Y., Chon Y.E., Lee J.H., Simon T.G., Chan A.T., Hwang S.G. (2021). Body weight variability and the risk of cardiovascular outcomes in patients with nonalcoholic fatty liver disease. Sci. Rep..

[7] Kuchay M.S., Martinez-Montoro J.I., Choudhary N.S., Fernandez-Garcia J.C., Ramos-Molina B. (2021). Non-Alcoholic Fatty Liver Disease in Lean and Non-Obese Individuals: Current and Future Challenges. Biomedicines.

[8] Ye Q., Zou B., Yeo Y.H., Li J., Huang D.Q., Wu Y., Yang H., Liu C., Kam L.Y., Tan X.X. (2020). Global prevalence, incidence, and outcomes of non-obese or lean non-alcoholic fatty liver disease: A systematic review and meta-analysis. Lancet Gastroenterol. Hepatol..

[9] Sookoian S., Pirola C.J. (2017). Systematic review with meta-analysis: Risk factors for non-alcoholic fatty liver disease suggest a shared altered metabolic and cardiovascular profile between lean and obese patients. Aliment. Pharmacol. Ther..

[10] Young S., Tariq R., Provenza J., Satapathy S.K., Faisal K., Choudhry A., Friedman S.L., Singal A.K. (2020). Prevalence and Profile of Nonalcoholic Fatty Liver Disease in Lean Adults: Systematic Review and Meta-Analysis. Hepatol. Commun..

[11] Tan E.X., Lee J.W., Jumat N.H., Chan W.K., Treeprasertsuk S., Goh G.B., Fan J.G., Song M.J., Charatcharoenwitthaya P., Duseja A. (2021). Non-obese non-alcoholic fatty liver disease (NAFLD) in Asia: An international registry study. Metabolism.

[12] Niriella M.A., Kasturiratne A., Pathmeswaran A., De Silva S.T., Perera K.R., Subasinghe S.K., Kodisinghe S.K., Piyaratna T.A., Vithiya K., Dassanayaka A.S. (2019). Lean non-alcoholic fatty liver disease (lean NAFLD): Characteristics, metabolic outcomes and risk factors from a 7-year prospective, community cohort study from Sri Lanka. Hepatol. Int..

[13] Eslam M., Chen F., George J. (2020). NAFLD in Lean Asians. Clin. Liver Dis..

[14] Yang K.C., Hung H.F., Lu C.W., Chang H.H., Lee L.T., Huang K.C. (2016). Association of Non-alcoholic Fatty Liver Disease with Metabolic Syndrome Independently of Central Obesity and Insulin Resistance. Sci. Rep..

[15] Sookoian S., Pirola C.J. (2018). Systematic review with meta-analysis: The significance of histological disease severity in lean patients with nonalcoholic fatty liver disease. Aliment. Pharmacol. Ther..

[16] History of Siriraj Hospital Faculty of Medicine Siriraj Hospital. https://www.si.mahidol.ac.th/sirirajhospital/history.php.

[17] Huang P.L. (2009). A comprehensive definition for metabolic syndrome. Dis. Model Mech..

[18] Tan C.E., Ma S., Wai D., Chew S.-K., Tai E.-S. (2004). Can We Apply the National Cholesterol Education Program Adult Treatment Panel Definition of the Metabolic Syndrome to Asians?. Diabetes Care.

[19] Grundy S.M., Cleeman J.I., Daniels S.R., Donato K.A., Eckel R.H., Franklin B.A., Gordon D.J., Krauss R.M., Savage P.J., Smith S.C. (2005). Diagnosis and management of the metabolic syndrome: An American Heart Association/National Heart, Lung, and Blood Institute Scientific Statement. Circulation.

[20] European Association for the Study of the Liver, European Association for the Study of Diabetes, European Association for the Study of Obesity (2016). EASL-EASD-EASO Clinical Practice Guidelines for the management of non-alcoholic fatty liver disease. J. Hepatol..

[21] Dumitrascu D.L., Neuman M.G. (2018). Non-alcoholic fatty liver disease: An update on diagnosis. Clujul Med..

[22] Puri P., Sanyal A.J. (2012). Nonalcoholic fatty liver disease: Definitions, risk factors, and workup. Clin. Liver Dis..

[23] European Association for the Study of the Liver (2021). Electronic address eee. EASL Clinical Practice Guidelines on non-invasive tests for evaluation of liver disease severity and prognosis—2021 update. J. Hepatol..

[24] Machado M.V., Cortez-Pinto H. (2013). Non-invasive diagnosis of non-alcoholic fatty liver disease. A critical appraisal. J. Hepatol..

[25] Jinjuvadia R., Antaki F., Lohia P., Liangpunsakul S. (2017). The Association Between Nonalcoholic Fatty Liver Disease and Metabolic Abnormalities in The United States Population. J. Clin. Gastroenterol..

[26] Rahman M.M., Kibria M.G., Begum H., Haque M., Sultana N., Akhter M., Rowshon A.H., Ahmed F., Hasan M. (2020). Prevalence, risk factors and metabolic profile of the non-obese and obese non-alcoholic fatty liver disease in a rural community of South Asia. BMJ Open Gastroenterol..

[27] Goyal A., Arora H., Arora S. (2020). Prevalence of fatty liver in metabolic syndrome. J. Family Med. Prim Care.

[28] Shaikh M.A., Bhanuprakash P. (2018). Study of non-alcoholic fatty liver disease in metabolic syndrome. Int. J. Adv. Med..

[29] Eslam M., Sanyal A.J., George J., Sanyal A., Neuschwander-Tetri B., Tiribelli C., Kleiner D.E., Brunt E., Bugianesi E., Yki-Järvinen H. (2020). MAFLD: A Consensus-Driven Proposed Nomenclature for Metabolic Associated Fatty Liver Disease. Gastroenterology.

[30] Younossi Z.M., Stepanova M., Negro F., Hallaji S., Younossi Y., Lam B., Srishord M. (2012). Nonalcoholic fatty liver disease in lean individuals in the United States. Medicine.

[31] Lu F.B., Zheng K.I., Rios R.S., Targher G., Byrne C.D., Zheng M.H. (2020). Global epidemiology of lean non-alcoholic fatty liver disease: A systematic review and meta-analysis. J. Gastroenterol. Hepatol..

[32] Lonardo A., Nascimbeni F., Ballestri S., Fairweather D., Win S., Than T.A., Abdelmalek M.F., Suzuki A. (2019). Sex Differences in Nonalcoholic Fatty Liver Disease: State of the Art and Identification of Research Gaps. Hepatology.

[33] Masarone M., Rosato V., Aglitti A., Bucci T., Caruso R., Salvatore T., Sasso F.C., Tripodi M.F., Persico M. (2017). Liver biopsy in type 2 diabetes mellitus: Steatohepatitis represents the sole feature of liver damage. PLoS ONE.

[34] Rinaldi L., Pafundi P.C., Galiero R., Caturano A., Morone M.V., Silvestri C., Giordano M., Salvatore T., Sasso F.C. (2021). Mechanisms of Non-Alcoholic Fatty Liver Disease in the Metabolic Syndrome. A Narrative Review. Antioxidants.

[35] Galiero R., Caturano A., Vetrano E., Cesaro A., Rinaldi L., Salvatore T., Marfella R., Sardu C., Moscarella E., Gragnano F. (2021). Pathophysiological mechanisms and clinical evidence of relationship between Nonalcoholic fatty liver disease (NAFLD) and cardiovascular disease. Rev. Cardiovasc. Med..

[36] Adinolfi L.E., Petta S., Fracanzani A.L., Nevola R., Coppola C., Narciso V., Rinaldi L., Calvaruso V., Pafundi P.C., Lombardi R. (2020). Reduced incidence of type 2 diabetes in patients with chronic hepatitis C virus infection cleared by direct-acting antiviral therapy: A prospective study. Diabetes Obes. Metab..

[37] Adinolfi L.E., Petta S., Fracanzani A.L., Coppola C., Narciso V., Nevola R., Rinaldi L., Calvaruso V., Staiano L., Di Marco V. (2020). Impact of hepatitis C virus clearance by direct-acting antiviral treatment on the incidence of major cardiovascular events: A prospective multicentre study. Atherosclerosis.

[38] Sasso F.C., Pafundi P.C., Caturano A., Galiero R., Vetrano E., Nevola R., Petta S., Fracanzani A.L., Coppola C., Di Marco V. (2021). Impact of direct acting antivirals (DAAs) on cardiovascular events in HCV cohort with pre-diabetes. Nutr. Metab. Cardiovasc. Dis..

[39] Kwon Y.M., Oh S.W., Hwang S.S., Lee C., Kwon H., Chung G.E. (2012). Association of nonalcoholic fatty liver disease with components of metabolic syndrome according to body mass index in Korean adults. Am. J. Gastroenterol..

[40] Li Y., Chen Y., Tian X., Zhang S., Jiao J. (2021). Comparison of Clinical Characteristics Between Obese and Non-Obese Patients with Nonalcoholic Fatty Liver Disease (NAFLD). Diabetes Metab. Syndr. Obes..

[41] Hadizadeh F., Faghihimani E., Adibi P. (2017). Nonalcoholic fatty liver disease: Diagnostic biomarkers. World J. Gastrointest. Pathophysiol..

[42] Alam S., Eslam M., SKMHasan N., Anam K., Chowdhury M.A., Khan M.A., Hasan M.J., Mohamed R. (2021). Risk factors of nonalcoholic fatty liver disease in lean body mass population: A systematic review and meta-analysis. JGH Open.

[43] Khayyat Y.M. (2021). Determination of “indeterminate score” measurements in lean nonalcoholic fatty liver disease patients from western Saudi Arabia. World J. Hepatol..

[44] Saokaew S., Kositamongkol C., Charatcharoenwitthaya P., Srivanichakorn W., Washirasaksiri C., Chaiyakunapruk N., Phisalprapa P. (2020). Comparison of noninvasive scoring systems for the prediction of nonalcoholic fatty liver disease in metabolic syndrome patients. Medicine.

[45] Hsu C.L., Wu F.Z., Lin K.H., Chen Y.H., Wu P.C., Chen Y.H., Chen C.S., Wang W.H., Mar G.Y., Yu H.C. (2019). Role of Fatty Liver Index and Metabolic Factors in the Prediction of Nonalcoholic Fatty Liver Disease in a Lean Population Receiving Health Checkup. Clin. Transl. Gastroenterol..

[46] Hernaez R., Lazo M., Bonekamp S., Kamel I., Brancati F.L., Guallar E., Clark J.M. (2011). Diagnostic accuracy and reliability of ultrasonography for the detection of fatty liver: A meta-analysis. Hepatology.

[47] Castera L., Forns X., Alberti A. (2008). Non-invasive evaluation of liver fibrosis using transient elastography. J. Hepatol..

[48] Xiao G., Zhu S., Xiao X., Yan L., Yang J., Wu G. (2017). Comparison of laboratory tests, ultrasound, or magnetic resonance elastography to detect fibrosis in patients with nonalcoholic fatty liver disease: A meta-analysis. Hepatology.

